# Chronic Radiation-Induced Wounds: Pathogenesis, Current Therapeutic Strategies, and Emerging Regenerative Approaches

**DOI:** 10.3390/ijms27188221

**Published:** 2026-09-15

**Authors:** Luyao Yu, Shangfei Gao, Cuicui Han, Jing Zhang, Jiaxu Chen, Xiaowen Xing, Beier Jiang, Zheng Zhou, Yifei Feng, Ying He

**Affiliations:** 1 Navy Medical Centre, Naval Medical University, Shanghai 200433, China; 2 College of Food Science and Technology, Shanghai Ocean University, Shanghai 201306, China

**Keywords:** radiation-induced chronic wounds, oxidative stress, fibrosis, exosomes, regenerative medicine

## Abstract

Radiation-induced chronic wounds remain a major clinical challenge owing to persistent non-healing and progressive tissue degeneration. Rather than simply representing delayed wound repair, they arise from a self-perpetuating pathological cascade involving DNA damage, oxidative stress, chronic inflammation, microvascular dysfunction, cellular senescence, stem cell impairment, and fibrosis. Current clinical management remains largely supportive and symptomatic, with limited efficacy and no standardized therapeutic regimens targeting the core pathological mechanisms. Emerging strategies—antioxidant, antifibrotic, senolytic, stem cell/exosome-based, and functional biomaterial approaches—show promise. This review critically summarizes recent advances in pathogenic mechanisms and mechanism-based therapeutic strategies, highlighting current translational challenges and future directions for microenvironment-oriented regenerative therapies.

## 1. Introduction

Ionizing radiation is widely used in cancer radiotherapy, interventional radiology, and the nuclear industry. However, unintended radiation exposure can cause radiation-induced skin injury (RISI), an umbrella term that encompasses acute and late cutaneous effects [1,2]. In this review, chronic radiation dermatitis refers to late cutaneous manifestations of RISI, including atrophy, telangiectasia, pigmentary changes, and fibrosis; ulceration may occur in severe cases. We use radiation-induced chronic wounds (RICWs) as the primary term for persistent, non-healing ulcerative lesions arising in irradiated skin. The term radiation ulcer is used only when referring specifically to an ulcerative lesion or to the terminology used in the cited literature. RICWs are characterized by persistent non-healing, recurrent ulceration, progressive fibrosis, microvascular dysfunction, and an increased risk of infection and malignant transformation. Unlike acute mechanical or thermal injuries, which generally undergo an orderly sequence of inflammation, proliferation, and remodeling, radiation-induced chronic wounds arise from sustained tissue damage and dysregulated repair processes [3]. Accumulating evidence suggests that their development involves a complex pathological cascade initiated by DNA damage and oxidative stress and perpetuated by chronic inflammation, vascular impairment, cellular senescence, stem cell exhaustion, and fibrotic remodeling. As a result, affected tissues remain trapped in a persistent non-healing state that is difficult to reverse [4].

Despite advances in wound care and radiation protection, current clinical management remains largely supportive and includes anti-inflammatory treatment, growth-factor supplementation, infection control, and local wound care. Although these approaches may alleviate symptoms and support local healing, their ability to modify the persistent vascular, cellular, and fibrotic abnormalities underlying established radiation-induced chronic wounds remains limited. Accordingly, standardized mechanism-based therapies have yet to be established [5,6]. Recent progress in radiation biology, regenerative medicine, and biomaterials research has provided new insights into the pathogenesis of radiation-induced chronic wounds and has identified several promising therapeutic targets. In particular, interventions aimed at oxidative stress, cellular senescence, fibrosis, stem cell dysfunction, and microvascular injury are emerging as potential avenues for disease modification and functional tissue regeneration.

While recent comprehensive reviews have significantly advanced our understanding of complex wound chronicity, they have predominantly emphasized biofilm-mediated recalcitrance in generic ulcers [7,8] or metabolic impairments in diabetic models [9]. Similarly, contemporary molecular explorations of radiation pathology have largely concentrated on internal organ systems, such as the liver [10], leaving a critical knowledge gap regarding cutaneous-specific radiation injuries. This review distinguishes itself by shifting the analytical focus away from generic microbial or systemic metabolic impediments to decode the unique, deep-seated radiopathology of the skin. By uniquely consolidating recent single-cell transcriptomic and epigenetic discoveries, we elucidate the irreversible “profibrotic memory,” stem cell exhaustion, and microvascular obliteration that exclusively define these lesions. By integrating recent single-cell transcriptomic and epigenetic findings, this review examines how persistent alterations in cellular, vascular, and extracellular-matrix microenvironments may inform the development of targeted regenerative strategies.

This review summarizes the clinical characteristics, pathological progression, molecular mechanisms, and current therapeutic strategies of radiation-induced chronic wounds, with particular emphasis on emerging targeted therapies and regenerative approaches. By integrating recent basic and translational evidence, we aim to provide a framework for understanding disease pathogenesis and for guiding future therapeutic development.

## 2. Clinical Characteristics and Pathological Mechanisms of Radiation-Induced Chronic Wounds

The persistence of these refractory wounds stems directly from a profound disruption of normal tissue homeostasis. While the fundamental molecular mechanisms underlying radiation-induced chronic wounds are largely qualitative in nature, integrating specific epidemiological and clinical case data provides indispensable quantitative evidence for their clinical relevance and pathological progression [11,12,13]. Late RISI, including chronic radiation dermatitis and RICWs, may emerge months to decades after irradiation. Chronic radiation dermatitis has been reported from approximately 90 days to as long as 20–30 years after treatment [12,13,14].

Importantly, clinical series of ulcerative RICWs, often termed radiation-induced ulcers in the original reports, illustrate their prolonged and refractory course. In a retrospective series of 64 patients with chronic radiation-induced chest-wall ulcers following breast cancer treatment, ulcers developed 3–24 years after radiotherapy (mean, 11 years), and ulcer severity showed a significant dose-dependent relationship (*p* < 0.001) [15]. Similarly, a prospective series of 30 patients undergoing reconstruction for radiation-induced ulcers reported a median interval of 7.5 years between radiotherapy and ulcer development (range, 1–31 years), with the chest being the most frequently affected site [16].

In another 10-year retrospective study of 50 women with chronic radiation-induced chest-wall ulcers after breast cancer radiotherapy, all patients required reconstructive surgery, underscoring the refractory nature of established ulcerative RICWs [17]. Although the incidence of chronic radiation ulcers varies substantially according to radiation dose, irradiated volume, treatment technique, anatomical site, and patient-related factors, a retrospective study of 612 irradiated skin fields reported radiogenic ulcers in 58 fields (9.4%) after long-term follow-up, with ulcer frequency increasing with radiation dose.

Chronic radiation injury is also associated with an increased risk of secondary cutaneous malignancy. Available clinical data indicate that secondary skin cancers are an important but variably reported late complication, with squamous cell carcinoma representing a recognized malignant outcome of chronic radiation-damaged skin.

These quantitative clinical observations demonstrate that radiation-induced chronic wounds are not merely theoretical consequences of molecular injury, but clinically evident, delayed, and often refractory complications that can substantially impair quality of life [11,12,14]. Consequently, the following subsections dissect the molecular hallmarks driving these clinical manifestations, thereby bridging observed clinical outcomes with their underlying pathological mechanisms.

Rather than a mere kinetic delay of canonical wound healing, radiation-induced recalcitrant cutaneous injury represents a systemic, irreversible pathophysiological derailment spanning from genomic instability and cellular senescence to macro-tissue microenvironmental collapse. The crux of this pathogenesis lies in the capacity of ionizing radiation to propagate multifaceted cascade reactions that fundamentally shatter the essential pillars of cutaneous regeneration: the resident stem cell reservoir, the microvascular network, and immune homeostasis. The overarching pathological progression unfolds through a tripartite chronological cascade: acute injury initiation, chronic inflammatory maintenance, and fibrotic remodeling coupled with tissue exhaustion. These consecutive phases do not exist in isolation but actively cross-talk to form a self-amplifying vicious cycle, collectively constituting the core driving mechanism behind the protracted, non-healing nature of radiation-induced wounds.

**Figure 1:**
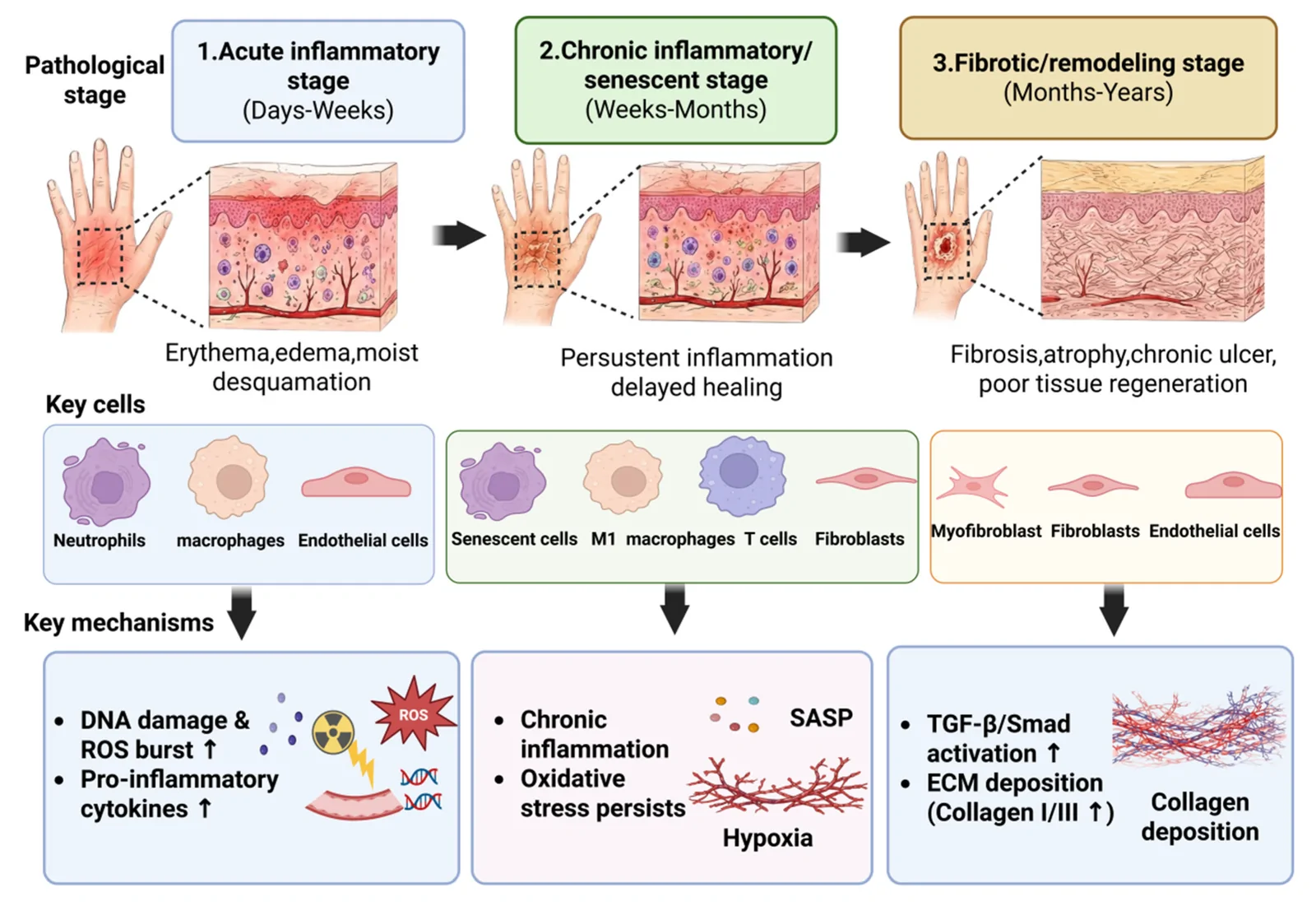
Graphical overview of the three-stage pathological cascade in radiation-induced chronic wounds. Blue, green, and yellow/orange panels represent the acute inflammatory, chronic inflammatory/senescent, and fibrotic/remodeling stages, respectively. Thick arrows indicate pathological progression or causal relationships; small ↑ arrows denote increased and decreased levels or activity, respectively.

To provide a concise overview of the major cellular, molecular, and microenvironmental events driving the progression from acute RISI to late-stage RICWs, we summarize the key features of each pathological stage in Figure 1. The following Section 2.1, Section 2.2 and Section 2.3 discuss the underlying mechanisms of each phase in detail.

### 2.1. Acute Injury Initiation: Genomic Instability and Oxidative Stress Triggering the Inflammatory Cascade

Upon traversing cutaneous tissues, ionizing radiation exerts its primary, immediate damage directly on the nuclear DNA and the intracellular redox homeostasis system. Radiant energy directly induces diverse types of genomic lesions, including DNA single-strand breaks and base modifications, with DNA double-strand breaks (DSBs) standing out as the most severe form of genotoxic insult. Upon occurrence, DSBs promptly activate the ATM/ATR kinase-mediated DNA damage response (DDR), which subsequently propagates downstream p53 and CHK1/CHK2 signaling pathways to orchestrate cell cycle arrest, DNA repair, or apoptotic programs [18,19,20]. When the radiation dose overwhelms the intrinsic cellular repair capacity, the persistence and accumulation of unrepaired or misrepaired DNA lesions lead to the release of cytosolic DNA, which activates the cGAS-STING pathway. This innate immune axis, in turn, modulates the DNA damage response and promotes inflammatory signaling. Ultimately, the persistent DNA lesions serve as a critical instigator of subsequent cellular senescence, mutations, and functional aberrations [21]. Persistent or misrepaired DNA damage may promote cellular senescence and reduce the proliferative capacity of irradiated resident cells. These changes may limit the responsiveness of established radiation-induced wounds to therapies that primarily depend on endogenous cellular proliferation, particularly in severely damaged tissue; however, treatment responses are likely to vary according to radiation dose, wound stage, residual tissue viability, and adjunctive interventions.

Concurrently, ionizing radiation drives the radiolysis of intracellular water molecules, triggering a massive surge of reactive oxygen species (ROS) such as superoxide anions, hydroxyl radicals, and hydrogen peroxide [22]. Possessing potent oxidative reactivity, these ROS directly assault cell membrane lipids, intracellular proteins, and genomic DNA, triggering lipid peroxidation, protein carbonylation, and oxidative DNA lesions. This comprehensive oxidative assault further exacerbates genomic instability and destabilizes cellular functional machinery. Under physiological conditions, cells maintain redox homeostasis and scavenge ROS via endogenous antioxidant enzymes—including superoxide dismutase (SOD), glutathione peroxidase (GPx), and heme oxygenase-1 (HO-1)—coupled with the Nrf2 (nuclear factor erythroid 2-related factor 2) antioxidant pathway [23,24]. Following radiation, however, the catastrophic ROS burst thoroughly overwhelms the scavenging capacity of this intrinsic defense machinery; the subsequent hyperactivation and eventual exhaustion of the Nrf2 signaling axis lead to a state of unmitigated, persistent oxidative stress, which serves as a pivotal driver propagating subsequent inflammatory responses and propagating tissue damage [25]. Unlike the transient hypoxia seen in standard chronic wounds, this radiation-induced continuous radical generation creates a permanently toxic microenvironment. Persistent ROS generation may help explain why exogenous antioxidant approaches show variable or limited benefit in some settings. However, direct comparative clinical evidence evaluating their efficacy in established radiation-induced chronic wounds remains limited. Operating synergistically, genomic damage and oxidative stress drive programmed cell death, liberating damage-associated molecular patterns (DAMPs) such as HMGB1 and ATP. These danger signals are recognized by surface receptors (e.g., TLR4, RAGE), hyperactivating NF-κB, MAPK, and NLRP3 inflammasome pathways to trigger a catastrophic pro-inflammatory cytokine storm [26,27]. In response, resident cutaneous structural cells—including keratinocytes, fibroblasts, and vascular endothelial cells—take the lead in secreting pro-inflammatory cytokines such as interleukin-1β (IL-1β), tumor necrosis factor-α (TNF-α), and interleukin-6 (IL-6). Concurrently, they release pivotal chemokines, prominently CXC chemokine ligand 2 (CXCL2) and monocyte chemoattractant protein-1 (MCP-1), to rapidly mobilize and recruit circulating immune cells, such as neutrophils and macrophages, to infiltrate the site of injury [28,29,30].

Once overactivated, these infiltrated neutrophils release neutrophil extracellular traps (NETs) to further exacerbate local tissue destruction, while macrophages predominantly assume a pro-inflammatory M1 phenotype during this early stage, continuously amplifying inflammatory signals to propagate a localized acute inflammatory storm [31].

In addition to apoptosis and pyroptosis, ionizing radiation can trigger ferroptosis, an iron-dependent form of regulated cell death characterized by excessive lipid peroxidation. Radiation-induced ROS generation, together with disrupted iron homeostasis, promotes lipid peroxidation, while suppression of the SLC7A11 (System L amino acid transporter light chain subunit)–GSH–GPX4 antioxidant axis further compromises cellular protection against lipid peroxides [32].

Recent evidence directly implicates ferroptosis in radiation-induced skin injury, with increased labile iron, lipid peroxidation, and ferroptosis-associated molecular changes following irradiation. In a preclinical model, transdermal deferoxamine (DFO) improved excisional wound healing in chronically irradiated murine skin, supporting the therapeutic potential of iron chelation in radiation-damaged tissues [33]. Ferroptotic cells can also release DAMPs and activate innate immune signaling, thereby linking redox imbalance to sustained inflammation [34].

Importantly, ferroptosis has been implicated in radiation-induced fibrotic remodeling, as inhibition of ferroptosis reduces TGF-β1 expression and attenuates radiation-induced fibrosis. Recent single-cell analyses further suggest that ferroptosis-associated pathways are enriched in myofibroblasts and correlate with extracellular matrix remodeling during radiation-induced fibrosis [35]. Together, these findings position ferroptosis as a potential mechanistic bridge linking acute radiation-induced oxidative injury and chronic inflammatory–fibrotic remodeling. From a critical perspective, this cascade represents a self-sustaining sterile inflammatory loop rather than a physiological healing response. Therefore, broad-spectrum anti-inflammatory agents may provide symptomatic benefit but may be insufficient, when used alone, to address the multiple DAMP- and SASP-associated processes implicated in persistent tissue injury. Whether these mechanisms account for long-term clinical outcomes in established radiation-induced chronic wounds requires further study. Ultimately, although the pathological manifestations initially present as acute inflammation, the sustained transduction of genomic damage, oxidative stress, and inflammatory signaling trajectories insidiously lays the pathological groundwork for the subsequent maintenance of chronic inflammation and stalled healing kinetics.

**Figure 2:**
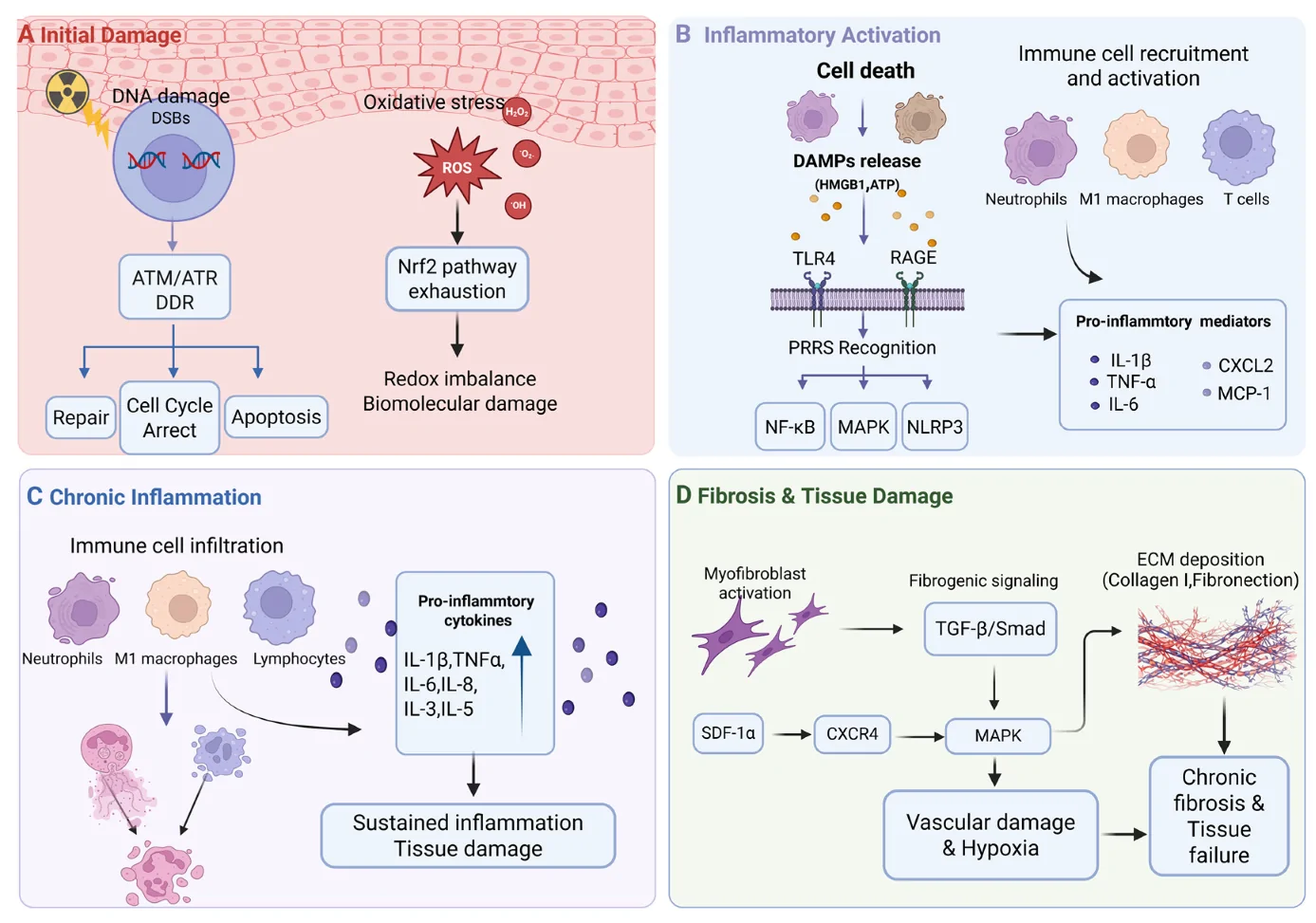
Acute injury initiation: DNA damage, oxidative stress, and inflammatory cascade in radiation-induced skin injury. (**A**) DNA double-strand breaks (DSBs) activate the ATM/ATR DDR pathway, while ROS leads to Nrf2 exhaustion, redox imbalance, and biomolecular damage. (**B**) Cell death releases damage factors, activating NF-κB/MAPK/NLRP3 pathways to induce the release of pro-inflammatory mediators (IL-1β, TNF-α) and recruit neutrophils, M1 macrophages, and T cells. (**C**) Persistent infiltration of inflammatory immune cells leads to the secretion of pro-inflammatory cytokines (IL-1β, TNF-α, IL-6, IL-8), resulting in sustained inflammation and progressive tissue damage. (**D**) Myofibroblast activation via TGF-β/Smad triggers ECM deposition, which is exacerbated by vascular damage and hypoxia, ultimately leading to chronic fibrosis and tissue failure. Note: Thick arrows indicate pathological progression or causal relationships; small ↑ arrows denote increased and decreased levels or activity, respectively. Color-coded panels distinguish the major pathological modules, while cellular and tissue patterns schematically illustrate the associated biological and histopathological changes.

The key molecular pathways initiating this acute inflammatory cascade are schematically summarized in Figure 2.

### 2.2. Chronic Inflammatory Perpetuation and Arrested Wound Healing Programs

The failed resolution of acute inflammation serves as the critical pathological checkpoint driving radiation-induced wounds into a chronic, non-healing state. Driven by the unrelenting persistence of radiation-induced DNA damage and oxidative stress, resident cutaneous structural cells are forced into a senescent state and exhibit a SASP, which functions as the core pathological engine perpetuating chronic inflammation. This creates a bidirectional vicious cycle: SASP factors drive local inflammation, while elevated cytokines and ROS trigger secondary senescence in adjacent cells [36].

These senescent keratinocytes and fibroblasts chronically hypersecrete a potent array of pro-inflammatory cytokines and proteases, including IL-6, IL-8, TNF-α, and matrix metalloproteinases (MMPs). This secretory profile exerts its damage through dual pathological arms: on one hand, it perpetually recruits circulating immune cells to sustain the localized inflammatory response; on the other hand, it actively degrades the extracellular matrix (ECM) and dismantles the tissue regenerative microenvironment, thereby establishing a self-perpetuating, chronic inflammatory loop [37,38]. A protease-rich microenvironment may reduce the local bioavailability of exogenous growth factors and could contribute to variable treatment responses. However, the extent to which proteolytic degradation limits growth-factor efficacy in established radiation-induced chronic wounds has not been determined in comparative clinical studies.

The dysregulated polarization of macrophages constitutes another core mechanism driving chronic inflammatory maintenance and stalled wound healing. During canonical wound healing, macrophages must undergo a precise phenotypic transition from an early pro-inflammatory M1 phenotype to a pro-resolving M2 phenotype; these M2 macrophages sequentially secrete essential regenerative factors—including interleukin-10 (IL-10), transforming growth factor-β (TGF-β), and vascular endothelial growth factor (VEGF)—to orchestrate granulation tissue formation, angiogenesis, and re-epithelialization, following radiation insult [39,40]. However, the persistent convergence of DAMPs, SASP factors, and oxidative stress chronically hyperactivates the NF-κB and MAPK pathways, which concurrently suppress the expression of trigger receptor expressed on myeloid cells 2 (TREM2) and severely impede the phenotypic polarization toward the M2 lineage. Consequently, the wound microenvironment is locked into an intractable state wherein pro-inflammatory M1 macrophages chronically dominate alongside a profound deficit in reparative M2 sub-populations, ultimately culminating in failed inflammatory resolution, a dearth of regenerative signaling, and a non-healing state characterized by dense inflammatory infiltration and pale, dormant granulation tissue [41,42]. From a therapeutic standpoint, this profound deficit in M2 sub-populations means the local immune system actively antagonizes repair. Thus, treatments must pivot from merely suppressing acute inflammation to actively reprogramming macrophage fate to break this developmental arrest.

The functional exhaustion of fibroblasts constitutes a pivotal cellular event underlying the arrest of the tissue repair program. As the cornerstone cellular component of granulation tissue, fibroblasts are chronologically responsible for proliferation, type III collagen synthesis, directed migration, and ECM remodeling. Following radiation exposure, fibroblasts suffer a severe decline in proliferative and migratory capacity. Crucially, these senescent fibroblasts exhibit a blunted responsiveness to TGF-β signaling, failing to activate downstream repair networks or synthesize properly organized collagen [43,44]. This comprehensive cellular collapse leaves the wound bed devoid of nascent granulation tissue. Consequently, bioengineered scaffolds or conventional skin grafts placed into this cellularly depleted void often fail to integrate, as the indispensable host-derived fibroblastic machinery is functionally paralyzed.

Impaired angiogenesis compounds this regenerative failure. Ionizing radiation directly induces vascular endothelial cell senescence and apoptosis, while chronic inflammation (e.g., TNF-α) downregulates essential angiogenic drivers like VEGF and Ang-1 [45,46]. Experimental studies of radiation-combined skin wound injury have associated endothelial dysfunction and persistent inflammation with delayed wound repair [47]. Because severe microvascular injury may include structural as well as functional abnormalities, adjunctive therapies such as hyperbaric oxygen may not fully restore vascular integrity when used alone. Their capacity to provide durable benefit in established radiation-induced chronic wounds, particularly in severely ischemic lesions, requires further clinical evaluation.

Radiation-induced disruption of the cutaneous barrier and local immune homeostasis may also perturb the skin microbiome, introducing an additional layer of dysregulation within the chronic wound microenvironment. Clinical studies have shown that radiotherapy can alter the composition and diversity of the cutaneous microbiota, with changes in microbial communities associated with the severity and persistence of radiation-induced dermatitis [48].

Such dysbiosis may impair normal host–microbe homeostasis and increase the relative abundance of opportunistic microorganisms, thereby providing persistent microbial stimuli to an already inflamed tissue. Microbial products and biofilm-associated pathogen-associated molecular patterns (PAMPs) can activate innate immune receptors, including TLRs, and further enhance NF-κB- and MAPK-dependent inflammatory signaling. In this context, dysbiosis may act not merely as a consequence of impaired barrier function, but as a secondary amplifier of chronic inflammation, reinforcing cytokine production and tissue damage.

Persistent microbial imbalance may also interfere with epithelial barrier restoration and re-epithelialization, thereby contributing to delayed wound closure and prolonged tissue repair. In severe cases, biofilm formation by opportunistic pathogens such as Staphylococcus aureus may further aggravate local inflammation, hypoxia, and tissue destruction [48,49]. Thus, radiation-induced dysbiosis acts not just as a bystander, but as a critical driver that feeds back into the local inflammatory and ischemic cascades, further cementing the recalcitrant nature of radiation wounds.

However, these pathological processes are not merely parallel and additive. Bian et al. (2024) revealed that radiation leaves a persistent epigenetic memory in discrete dermal fibroblast subsets, manifested as irreversible chromatin accessibility alterations at specific promoters like *THBS1* [26]. This latent memory is pathologically activated only upon wounding, leading to aberrant THBS1 overexpression that suppresses fibroblast migration and contraction. This profound epigenetic reprogramming underscores a critical clinical reality: irradiated tissue is fundamentally distinct from normal tissue even before ulceration occurs. Eradicating this “trauma memory” at the chromatin level may therefore be an essential prerequisite for truly curative interventions.

**Figure 3:**
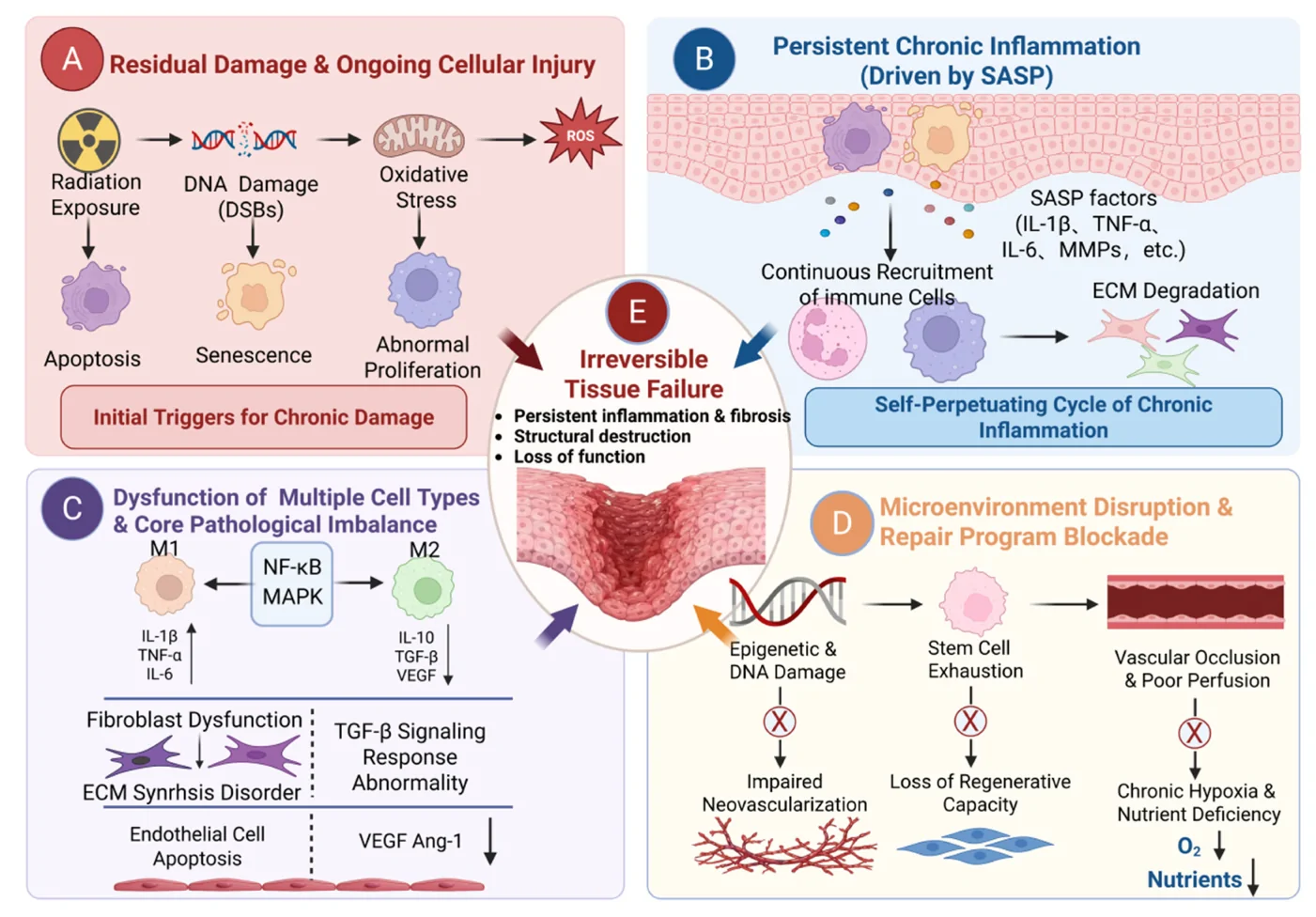
Chronic inflammatory perpetuation and arrested healing: cellular senescence, SASP, M1 macrophage polarization, and impaired angiogenesis. (**A**) Radiation exposure induces DNA damage and oxidative stress, establishing the initial triggers for chronic cellular injury. (**B**) Senescent cells activate the SASP, which drives continuous immune cell recruitment and ECM degradation, creating a self-perpetuating cycle of chronic inflammation. (**C**) Dysregulation of M1/M2 macrophage polarization leads to fibroblast dysfunction and endothelial cell apoptosis, resulting in core pathological imbalances. (**D**) Epigenetic and DNA damage, stem cell exhaustion, and vascular occlusion collectively block angiogenesis and regeneration, leading to tissue hypoxia and nutritional deprivation. (**E**) These interconnected pathological events culminate in persistent inflammation, fibrosis, and structural destruction, ultimately causing irreversible tissue failure. Note: Thick arrows indicate pathological progression or causal relationships; small ↑ and ↓ arrows denote increased and decreased levels or activity, respectively. Color-coded panels distinguish the major pathological modules, while cellular and tissue patterns schematically illustrate the associated biological and histopathological changes.

The interconnected pathological drivers that sustain chronic inflammation and impair tissue regeneration are schematically depicted in Figure 3.

### 2.3. Fibrotic Remodeling and Irreversible Tissue Exhaustion

Furthermore, this state is driven by intricate epigenetic landscapes—encompassing chromatin remodeling, DNA methylation anomalies, and aberrant histone modifications—which orchestrate a profound increase in accessibility at the promoter regions of fibrotic-associated genes such as *THBS1* to perpetuate excessive collagen deposition [50,51]. Critically, because this fibrotic trajectory is epigenetically locked and self-sustaining, interventions that merely target upstream inflammatory triggers are futile; therapeutic strategies must actively reverse this chromatin-level “profibrotic memory” to halt relentless scarring.

Single-cell RNA sequencing (scRNA-seq) further revealed the heterogeneous landscape of cellular responses in irradiated skin [52]. This study identified multiple cell subpopulations in irradiated human and murine skin, showing that radiation increased the proportions of fibroblasts and endothelial cells but reduced that of keratinocytes. Crucially, fibroblasts orchestrate extensive intercellular crosstalk, with specific subsets overexpressing targets like Nur77 to regulate localized radiosensitivity, macrophage interactions, and subsequent fibrotic trajectories. This profound cellular heterogeneity highlights a major limitation in current clinical paradigms: treating the irradiated wound as a uniform entity with broad-spectrum agents ignores subset-specific dysfunctions, underscoring the urgent need for precision interventions tailored to distinct cellular subpopulations.

In the context of radiation-induced imbalance in cellular composition and intercellular communication, the sustained hyperactivation of the TGF-β/Smad signaling axis constitutes the molecular centerpiece governing fibrotic remodeling. Chronically hypersecreted by infiltrating immune cells and senescent structural populations, TGF-β continuously stimulates the Smad2/3 signaling cascade, driving the transdifferentiation of resident fibroblasts into contractile myofibroblasts and triggering exorbitant synthesis of extracellular matrix (ECM) components such as type I collagen and fibronectin [53,54]. Concurrently, TGF-β suppresses the enzymatic activity of matrix metalloproteinases (MMPs) while upregulating tissue inhibitors of metalloproteinases (TIMPs), which severely blunts collagen degradation and further accelerates fibrotic deposition [55]. The resulting dense fibrotic matrix mechanically compresses the surrounding microvasculature, exacerbating localized hypoxia and stabilizing HIF-1α, which further fuels TGF-β and ROS production [56]. From a clinical standpoint, this dense structural rigidification establishes a formidable physical barrier to drug delivery and oxygen diffusion, rendering systemic pharmacological treatments largely ineffective and locking the tissue into a terminal “hypoxia-fibrosis” loop.

The irreversible exhaustion of epidermal stem cells (EpSCs), which are exquisitely radiosensitive, represents the root cause of permanent regenerative failure. Ionizing radiation profoundly disrupts this stem cell reservoir by triggering apoptotic cascades, cellular senescence, and aberrant asymmetric division patterns, culminating in a progressive, unresolving depletion of the functional stem cell pool. Consequently, the intrinsic regenerative potential of the skin is permanently extinguished, accompanied by the irreversible destruction of cutaneous appendages, including hair follicles, sweat glands, and sebaceous glands. Stripped of its native EpSC source, the severely compromised epidermal barrier completely loses the cellular engine required to fuel re-epithelialization, rendering the chronic wound eternally recalcitrant to healing [57,58]. Because the native stem cell reservoir is physically eradicated rather than merely dormant, conventional therapies utilizing topical growth factors inherently lack target cells to stimulate. Consequently, true re-epithelialization cannot be achieved without external cellular replacement or advanced tissue-engineered grafts.

The progressive obliteration of the microvasculature further exacerbates terminal tissue exhaustion. The prolonged intersection of chronic inflammation and unrelenting fibrotic remodeling induces severe endarteritis, structural fibrosis, and hyaline degeneration within the vascular walls, culminating in progressive luminal stenosis and eventual occlusion. Consequently, the local tissue is permanently plunged into a state of severe ischemia and hypoxia, characterized by the absolute disruption of nutrient delivery and the unmitigated stagnation of cytotoxic metabolic wastes. This persistent bioenergetic starvation feeds back to paralyze reparative cellular functions while forcefully driving pro-inflammatory and pro-fibrotic signaling cascades. Ultimately, this structural collapse manifests clinically as prominent skin atrophy, severe scar contracture, and chronic, non-healing ulcers highly susceptible to recurrent infection, or even drives malignant transformation into squamous cell carcinoma, establishing an absolute, irreversible state of functional tissue demise [59,60,61]. This terminal architectural collapse dictates a grim clinical reality: without aggressive surgical debridement and deep microvascular reconstruction, the wound bed remains an ischemic, necrotic sink, permanently dooming any superficial or purely conservative management to inevitable failure.

**Figure 4:**
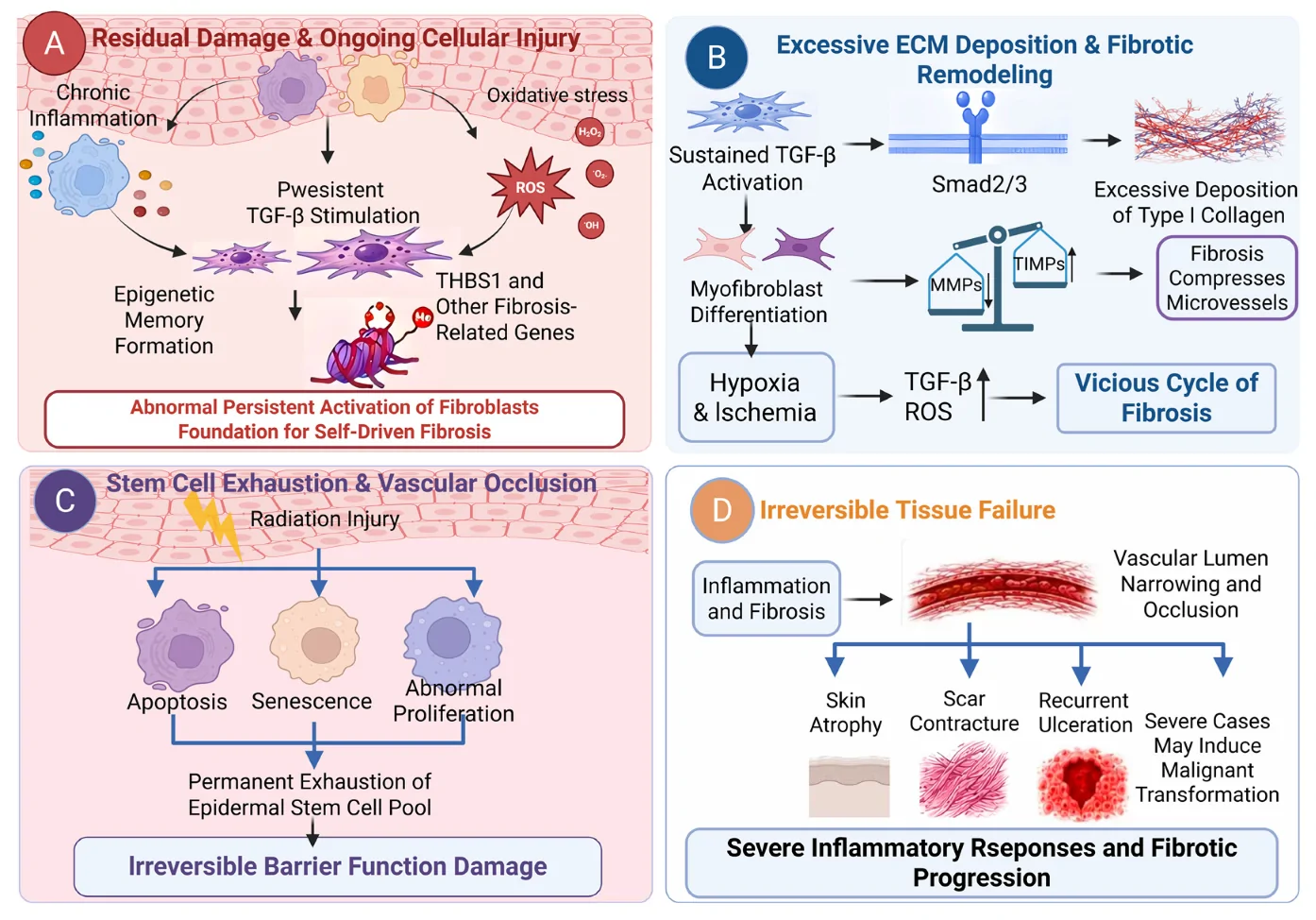
Fibrotic remodeling and terminal tissue exhaustion: TGF-β/Smad signaling, excessive ECM deposition, stem cell depletion, and microvascular occlusion. (**A**) Chronic inflammation and oxidative stress drive persistent TGF-β stimulation, leading to epigenetic changes and aberrant fibroblast activation, which lay the foundation for self-driven fibrosis. (**B**) Sustained TGF-β/Smad activation promotes myofibroblast differentiation and excessive collagen deposition, whereas ischemia/hypoxia exacerbates this fibrotic cycle and causes microvascular compression. (**C**) Radiation injury induces apoptosis, senescence, and abnormal proliferation of epidermal stem cells, causing permanent stem cell pool exhaustion and irreversible barrier function impairment. (**D**) Inflammation and fibrosis cause vascular lumen narrowing and occlusion, leading to skin atrophy, scar contracture, and recurrent ulceration, which may induce malignant transformation in severe cases. Note: Thick arrows indicate pathological progression or causal relationships; small ↑ and ↓ arrows denote increased and decreased levels or activity, respectively. Color-coded panels distinguish the major pathological modules.

The terminal pathological alterations that lead to irreversible fibrosis, stem cell exhaustion, and microvascular occlusion are schematically outlined in Figure 4.

In summary, radiation-induced non-healing wounds transition through a three-tiered irreversible pathological cascade: initiated by acute genotoxic damage and oxidative stress, sustained by chronic inflammatory perpetuation and stalled regenerative programs, and finalized by end-stage fibrotic remodeling and functional tissue exhaustion. These distinct yet overlapping phases mutually reinforce one another through vicious forward-looping feedback systems, which fundamentally dismantle the cellular, vascular, and microenvironmental foundations required for cutaneous repair, thereby rendering the refractory wound perpetually unresolving and behaviorally recalcitrant to conventional therapies. Ultimately, this multi-layered pathological collapse dictates that successful clinical intervention must transcend palliative symptom management, demanding precision multi-target strategies capable of concurrently restoring stem cell niches, reversing epigenetic memory, and rebuilding the microvascular network.

## 3. Clinical Manifestations and Current Therapeutic Landscape of Radiation-Induced Chronic Wounds

### 3.1. Clinical Presentation of Radiation-Induced Recalcitrant Cutaneous Lesions

RICWs represent a severe late manifestation of RISI. They typically arise in the context of chronic radiation dermatitis and are characterized by persistent non-healing, recurrent ulceration, impaired vascularization, fibrosis, increased susceptibility to infection, and a risk of malignant transformation [62]. Disease severity is compounded by radiation parameters (dose, field size, fractionation) and patient-specific comorbidities (diabetes, malnutrition, vascular disease), which synergistically compromise residual healing capacity [28]. Clinically, this protracted and unpredictable latency creates a deceptive therapeutic window; early conservative management often underestimates the insidious, progressive nature of the underlying tissue degradation, leading to delayed aggressive interventions until irreversible macroscopic necrosis has already been established.

The pathological progression of radiation-induced cutaneous injury typically spans a chronological spectrum composed of four distinct stages: the acute phase, the subacute phase, the chronic phase, and the late-stage fibrotic necrosis phase [63]. Each subsequent stage seamlessly transitions into the next with a progressive escalation in severity, thereby exhibiting a stereotypical pattern of stepwise tissue degradation; the specific clinical classifications and their corresponding manifestations are comprehensively delineated in Table 1. This shows that, rather than merely reacting to isolated symptoms at each phase, therapeutic strategies must anticipate and preemptively disrupt this continuous pathological continuum before terminal fibrotic exhaustion occurs.

In clinical oncology, the severity of radiation-induced cutaneous lesions is universally evaluated utilizing the Radiation Therapy Oncology Group (RTOG) scale or the Common Terminology Criteria for Adverse Events (CTCAE) grading system [1]. The scoring Tier functions as a direct prognostic indicator, wherein a higher grade strictly correlates with a more debilitating injury and a poorer clinical outcome; the granular parameters defining each grade are comprehensively indexed in Table 2.

Compared with conventional ulcers, radiation-induced chronic wounds exhibit a prolonged latency period, poor vascularization, marked fibrosis, frequent recurrence, increased susceptibility to infection, and a heightened risk of malignant transformation.

### 3.2. Current Therapeutic Status of Radiation-Induced Recalcitrant Wounds

The clinical management of radiation-induced chronic wounds remains challenging due to their complex and multifactorial pathophysiology. Current therapeutic strategies can be broadly categorized into four major groups: conventional symptomatic therapy, antioxidant and anti-fibrotic targeted therapy, regenerative medicine-based approaches, and physical adjunctive therapies. Each category targets distinct pathological levels, ranging from symptom control and inflammatory modulation to microenvironment reconstruction and tissue regeneration. Despite rapid advances in mechanistic understanding and therapeutic development, most interventions remain in early clinical or preclinical stages, and no standardized curative protocol has yet been established.

#### 3.2.1. Conventional Symptomatic Therapy

Conventional therapy remains the cornerstone of clinical management and primarily focuses on symptom relief, infection control, and superficial wound repair. This category includes local wound care, anti-inflammatory treatment, topical or systemic corticosteroids, non-steroidal anti-inflammatory drugs (NSAIDs), and exogenous growth factor supplementation.

Local wound care is fundamental in maintaining an appropriate wound microenvironment. It involves gentle cleansing using sterile saline or mild acidic solutions to minimize mechanical trauma to fragile granulation tissue and reduce microbial burden. Moisture balance is achieved using hydrating agents such as hydrogel, hyaluronic acid preparations, and petroleum-based ointments [64]. Depending on wound exudate levels, functional dressings including hydrocolloid, foam, and alginate dressings are applied to regulate exudate, protect the wound surface, reduce external irritation, and provide a stable environment for epithelial regeneration [65]. While local wound care may improve surface conditions and symptom control, it is primarily supportive and is not expected to directly reverse deeper vascular injury, fibrosis, or stem-cell dysfunction [66]. In established radiation-induced chronic wounds, local care alone may be insufficient for durable healing; however, the relative benefit of different wound-care strategies requires further comparative clinical evaluation.

Anti-inflammatory treatment, including corticosteroids (e.g., dexamethasone, hydrocortisone) and NSAIDs (e.g., ibuprofen, diclofenac sodium), acts primarily by inhibiting NF-κB- and MAPK-mediated inflammatory signaling pathways, thereby reducing acute inflammatory responses. However, prolonged use is associated with adverse effects such as skin atrophy, telangiectasia, and increased infection risk. However, evidence that these agents suppress persistent inflammation or prevent fibrotic progression in established radiation-induced chronic wounds is limited [67]. Their use is therefore generally directed toward symptom control or selected clinical indications, while the balance between potential benefit and adverse effects of prolonged treatment should be assessed individually.

Topical or recombinant growth factors, such as epidermal growth factor (EGF) and fibroblast growth factor (FGF), promote keratinocyte migration and superficial granulation tissue formation, thereby accelerating epithelial repair. Growth factors may support epithelial repair and granulation in selected wounds; however, their ability to modify deeper abnormalities, such as severe microvascular injury or stem-cell depletion, remains uncertain [68]. Recurrence may occur in established chronic lesions, although direct comparative clinical evidence in radiation-induced chronic wounds is limited. A high-ROS and protease-rich wound environment may further reduce growth-factor stability and bioavailability.

Overall, conventional therapies are accessible and clinically important for symptom control, infection management, and local wound care. However, their capacity to modify the underlying pathological processes in complex or advanced radiation-induced chronic wounds remains uncertain, and robust comparative evidence regarding durable healing and recurrence is limited.

#### 3.2.2. Antioxidant and Anti-Fibrotic Targeted Therapy

In recent years, targeted therapeutic strategies focusing on oxidative stress, cellular senescence, and fibrosis have become a central research focus. These approaches aim to intervene at key molecular checkpoints driving chronic wound persistence.

Antioxidant therapies primarily function by scavenging reactive oxygen species (ROS) and activating endogenous antioxidant pathways such as the Nrf2 signaling axis, thereby alleviating oxidative damage and reducing secondary inflammatory activation [69]. Representative agents include fullerols, selenium-based melanin compounds, and cordycepin. Among them, localized radioprotective hydrogel systems such as CPh-1014 have demonstrated selective protection of normal tissue without compromising tumor radiotherapy efficacy, showing promising early clinical outcomes [70]. Translation to established radiation-induced chronic wounds remains challenging because of issues such as in vivo stability, local retention, and maintenance of therapeutic concentrations within an oxidative wound environment. The clinical relevance of these proposed limitations requires further study.

Ferroptosis-targeted therapy represents an emerging strategy for mitigating radiation-associated tissue injury. Ferrostatin-1 and liproxstatin-1 are widely used experimental inhibitors of ferroptosis that suppress lipid peroxidation and interrupt iron-dependent cell death. Deferoxamine (DFO), an iron chelator, may reduce the labile iron pool and thereby limit ferroptotic injury. Preclinical studies indicate that pharmacological ferroptosis inhibition and iron chelation may mitigate radiation-associated tissue injury [32]. In particular, transdermal DFO improved excisional wound healing in chronically irradiated murine skin [33]. Notably, DFO-loaded silk fibroin hydrogels have been explored as a localized therapeutic strategy, combining ferroptosis suppression with pro-angiogenic effects in radiation-damaged tissues [71,72]. However, these approaches remain largely preclinical, and their optimal dosing, treatment timing, tissue specificity, and long-term safety require further investigation.

Anti-fibrotic strategies primarily target the TGF-β/Smad signaling pathway, which plays a central role in radiation-induced tissue fibrosis [73]. The combination of pentoxifylline and vitamin E has been widely used clinically to delay fibrotic progression [74]. In addition, statins, metformin, and artesunate have demonstrated anti-fibrotic potential in preclinical models through modulation of inflammatory and metabolic signaling pathways [75,76,77,78,79]. A significant challenge is the risk of off-target effects; since TGF-β is also vital for physiological repair, excessive inhibition may inadvertently impair residual tissue regeneration.

Senolytic therapies, such as the combination of dasatinib and quercetin, selectively eliminate senescent cells and suppress the senescence-associated secretory phenotype (SASP), thereby interrupting chronic inflammatory amplification loops and restoring tissue homeostasis [80,81]. Despite their potential, a critical concern remains whether clearing senescent cells might compromise the structural integrity of irradiated tissues or further exhaust local stem cell reserves.

Beyond the classic dasatinib and quercetin (D + Q) combination, various senolytic agents targeting distinct survival pathways have emerged [80,82]. The BCL-2/BCL-xL inhibitor navitoclax (ABT-263) has demonstrated potent senolytic activity by selectively eliminating senescent cells, and its therapeutic potential has been investigated in radiation-associated tissue injury and fibrosis [83,84]. In particular, senolytic treatment with ABT-263 has been shown to modulate the burden and function of senescent cells in radiation-induced skin injury, highlighting the potential of targeting persistent senescence in radiation-damaged tissues [85].

Similarly, the flavonoid fisetin exhibits senolytic activity against radiation-induced senescent cells in vitro, selectively reducing the viability of irradiated senescent endothelial cells [86]. HSP90 inhibitors, including 17-DMAG and ganetespib, represent another class of senolytic agents; notably, ganetespib selectively eliminated ionizing-radiation-induced senescent endothelial cells, while 17-DMAG showed senolytic activity across several cell types [87]. Collectively, these findings expand the potential therapeutic repertoire for targeting persistent senescence in chronic radiation-induced wounds. The feasibility of this strategy is further supported by evidence that D+Q treatment can reduce senescent-cell burden and mitigate ulcerative lesions in preclinical models of radiation-induced skin injury [80].

However, cellular senescence and senolysis may exert context- and phase-dependent effects during tissue repair [88]. Rather than being uniformly detrimental, transient senescence can play a physiological role in acute wound healing and tissue regeneration, whereas persistent senescence is more closely associated with chronic inflammation and impaired repair [89]. Senescent fibroblasts and endothelial cells can contribute to early wound closure through secretion of pro-reparative factors such as platelet-derived growth factor AA (PDGF-AA), which promotes myofibroblast differentiation and tissue repair. Importantly, this reparative role has been demonstrated directly in radiation-induced skin injury. Senescent fibroblasts accumulated during the reparative phase of radiation-induced skin injury and promoted re-epithelialization, collagen deposition, and angiogenesis, at least partly through secretion of interleukin-33 (IL-33) and modulation of macrophage polarization [85].

Conversely, genetic ablation of p16-positive senescent cells or treatment with senolytics, including ABT-263, delayed epidermal coverage, angiogenesis, and overall wound recovery. These findings indicate that premature or untimely elimination of senescent cells may disrupt beneficial reparative programs and paradoxically impair tissue regeneration [88,89].

Therefore, rather than adopting a one-size-fits-all clearance strategy, therapeutic targeting of senescence in radiation-induced chronic wounds should emphasize temporal and cell-type specificity. During the early reparative phase, transient senescent programs that support tissue remodeling, macrophage polarization, angiogenesis, and re-epithelialization may need to be preserved, whereas persistent, pro-inflammatory senescent cells accumulating during the chronic phase may represent more appropriate targets for senolytic intervention.

This distinction is consistent with the broader concept that transient senescence supports acute wound repair, whereas persistent senescence contributes to chronic wound pathology through sustained SASP-mediated inflammation, extracellular matrix dysregulation, and impaired regeneration [89].

Future studies should therefore focus on identifying pathological senescent cell populations according to their cellular identity, spatial distribution, and temporal dynamics, while developing targeted delivery systems capable of achieving precise spatial and temporal release of senolytic agents. Such an approach may maximize the therapeutic benefits of senolysis while minimizing disruption of physiologically beneficial senescence programs.

#### 3.2.3. Regenerative Medicine-Based Therapy

Regenerative medicine strategies aim to restore tissue homeostasis by reconstructing the wound microenvironment, enhancing angiogenesis, and reversing stem cell exhaustion. This category includes stem cell therapy, extracellular vesicle (exosome)-based therapy, and bioengineered biomaterials.

Mesenchymal stem cells (MSCs) exert therapeutic effects primarily through paracrine signaling, secreting growth factors, anti-inflammatory cytokines, and extracellular vesicles that collectively modulate immune responses, promote angiogenesis, and inhibit fibrosis [90,91]. Despite their broad regenerative potential, clinical translation is limited by challenges such as lack of standardized protocols, poor cell survival and retention in vivo, and high production costs.

Moreover, the wound microenvironment itself imposes additional constraints; Kusuma et al. (2017) reviewed that extracellular physicochemical cues—including oxygen tension, growth factors, and mechanical properties—critically determine MSC paracrine activity, and in hostile microenvironments such as those characterized by ischemia, chronic inflammation, and fibrotic matrix remodeling, the impaired MSC survival and secretory function further limit their therapeutic efficacy [92]. This hostile microenvironment may impair the survival and paracrine activity of transplanted cells, potentially limiting therapeutic efficacy; the extent of this effect in clinical radiation-induced chronic wounds remains to be established.

Although the hostile wound microenvironment may limit the survival and function of transplanted MSCs, several strategies have been explored to mitigate these constraints [93,94]. Preconditioning approaches, including hypoxic incubation, inflammatory-cytokine priming, and three-dimensional spheroid culture, have been reported to enhance MSC stress resistance and paracrine activity in experimental settings [93,95,96,97]. Additionally, genetic modification to overexpress anti-apoptotic factors (e.g., Bcl-2) or pro-angiogenic factors (e.g., VEGF) can further bolster MSC therapeutic efficacy in harsh environments [98].

Furthermore, biomaterial-based delivery systems, including hydrogels and scaffolds, provide protective microenvironments that enhance MSC survival, promote local retention, and support sustained paracrine activity within the wound bed [99,100]. Notably, the shift toward cell-free therapies using MSC-derived exosomes also represents a promising strategy to bypass the survival limitations of transplanted cells; however, optimizing their stability, targeting, and local retention still requires continued investigation [99,101,102].

Stem cell-derived exosomes have emerged as a promising cell-free therapeutic alternative. Enriched with microRNAs, proteins, and lipids, exosomes exhibit low immunogenicity, high stability, and scalable production potential. They can effectively suppress SASP activity, regulate inflammatory signaling, and enhance tissue repair processes, making them attractive candidates for clinical translation [103,104]. However, their clinical utility remains hampered by rapid systemic clearance and the lack of tissue-specific targeting to concentrate them at the lesion.

Bioengineered hydrogels, including cerium-based antioxidant hydrogels and exosome-loaded delivery systems, provide a three-dimensional scaffold that enables controlled release of bioactive molecules. These systems can simultaneously exert antioxidant, anti-inflammatory, and pro-angiogenic effects [105,106,107].

Recent advances in functional biomaterials, particularly hydrogel-based delivery systems, have demonstrated remarkable potential for the regeneration of radiation-induced wounds. Beyond the previously discussed cerium-based antioxidant hydrogels and exosome-loaded delivery systems [108], a diverse range of novel formulations has been developed to provide multifaceted reparative functions.

For instance, silk-based scaffolds, such as silk–fibrin composite gels and deferoxamine-loaded silk fibroin hydrogels, provide favorable biocompatibility, mechanical stability, and a supportive matrix for tissue regeneration. Importantly, the iron-chelating agent deferoxamine (DFO) can suppress radiation-induced ferroptosis while promoting angiogenesis [71,72].

Hydrogels incorporating antioxidant or immunomodulatory agents, including cannabidiol, curcumin, and tannic acid, have demonstrated antioxidant and anti-inflammatory activities and promoted wound repair in experimental radiation-associated skin-injury models [109,110].

Furthermore, oxygen-supplying hydrogels such as fibroin/perfluorocarbon hydrogels can directly alleviate local hypoxia and support cellular function and angiogenesis in radiation-associated tissue injury [111].

Notably, stimuli-responsive hydrogels (e.g., pH-, enzyme-, or ROS-sensitive systems) enable the on-demand release of therapeutic agents, providing a potential spatiotemporally controlled platform for stage-specific interventions [112,113].

Collectively, these advanced biomaterials serve not merely as physical wound covers, but as active microenvironment modulators, offering integrated solutions to the multifaceted challenges of oxidative stress, ferroptosis, hypoxia, and inflammation in radiation-induced tissue injury.

However, limitations remain regarding targeting specificity, long-term safety, and clinical standardization [114]. Furthermore, most hydrogels remain largely “passive” carriers that fail to mimic the dynamic, multi-modal signaling required for late-stage tissue remodeling. Overall, regenerative medicine represents a promising direction toward functional tissue restoration and potential cure; however, its clinical application is still constrained by delivery efficiency, manufacturing standardization, and regulatory challenges. Whether these strategies can produce durable clinical benefit in the presence of established ischemia and fibrosis remains uncertain and requires validation in well-designed clinical studies.

#### 3.2.4. Physical Adjunctive Therapy

Physical therapies are widely used as adjuvant approaches to improve local wound conditions and enhance the effectiveness of other treatments. These include hyperbaric oxygen therapy (HBOT), photodynamic therapy (PDT), infrared irradiation, and ultrasound-based interventions.

HBOT increases tissue oxygen tension and may improve ischemic conditions, thereby supporting wound healing in selected patients. Its use in radiation-associated tissue injury is generally considered adjunctive [115]. However, robust comparative evidence demonstrating durable benefit specifically in established radiation-induced chronic wounds remains limited, particularly in lesions with severe fibrosis or microvascular injury. Treatment-related risks, including oxygen toxicity, should also be considered in accordance with treatment protocols and individual patient factors.

Photobiomodulation (PBM), also known as low-level laser or low-level light therapy, is another promising physical modality for RISI. PBM uses red or near-infrared light to modulate mitochondrial bioenergetics and cellular redox signaling. Photons are absorbed primarily by mitochondrial cytochrome c oxidase (COX; complex IV), which enhances electron transport, mitochondrial membrane potential, and ATP production. This initial mitochondrial response generates a tightly controlled, transient increase in reactive oxygen species (ROS), acting as a crucial signaling intermediate rather than a source of oxidative damage to activate downstream cytoprotective and reparative pathways. Importantly, PBM exhibits a biphasic, dose-dependent response: appropriate light doses may promote mitochondrial function, antioxidant defense, cell proliferation, and tissue repair, whereas excessive irradiation may reduce or reverse these beneficial effects. Thus, the therapeutic efficacy of PBM depends strongly on wavelength, irradiance, fluence, treatment duration, and tissue context. In RISI, these effects may help restore mitochondrial function, modulate oxidative stress and inflammation, and support wound healing [116,117].

Clinical studies have reported beneficial effects of PBM in radiation-induced skin injury. Randomized controlled trials in patients receiving radiotherapy for breast or head and neck cancer have shown reductions in the severity of acute radiation dermatitis, particularly higher-grade skin reactions [118,119,120]. Importantly, PBM has also been reported in patients with established radiation-induced chronic ulcers and fistulae, suggesting potential utility beyond the prevention of acute radiodermatitis [121].

However, the clinical evidence remains heterogeneous, and treatment parameters—including wavelength, energy density, irradiation frequency, and treatment timing—vary substantially across studies. Whether PBM can effectively reverse advanced radiation-induced fibrosis, microvascular obliteration, or extensive tissue necrosis remains unclear. Thus, PBM should currently be regarded as a supportive adjunct rather than a definitive disease-modifying therapy.

PDT exerts antimicrobial effects through reactive oxygen species generation and has been shown to enhance wound healing by reducing infection burden and modulating local inflammatory responses [122,123]. However, generating ROS for antisepsis may inadvertently worsen the severe oxidative stress intrinsic to radiation injuries. Infrared and ultrasound therapies improve microcirculation, reduce inflammation, and promote tissue regeneration, offering safe and easily applicable adjunctive benefits [124]. Yet, their utility is constrained by limited tissue penetration and a lack of standardized energy dosing protocols.

Despite these advantages, the clinical translation of functional biomaterials remains constrained by insufficient standardization across multiple critical dimensions, including material composition, batch-to-batch reproducibility, mechanical performance, degradation and drug-release kinetics, sterilization methods, shelf-life stability, and biocompatibility evaluation. These challenges are particularly relevant to multifunctional hydrogels, in which crosslinking chemistry, bioactive components, and sterilization procedures can substantially alter material properties and therapeutic performance.

In addition, scalable GMP-compliant manufacturing and predefined quality-control and product-release criteria remain insufficiently established for many experimental systems. Standardized characterization protocols, validated sterilization procedures, reproducible manufacturing processes, and harmonized preclinical and regulatory evaluation criteria will therefore be essential for advancing these materials from proof-of-concept studies toward clinical application.

To provide a comprehensive overview and facilitate direct comparison, the key characteristics, primary mechanisms, inherent advantages, and current translational challenges of the emerging therapeutic strategies discussed above are summarized in Table 3.

### 3.3. Ongoing Clinical Studies and Translational Landscape

Despite substantial progress in preclinical research, the clinical translation of emerging therapies for radiation-induced tissue injury remains limited. Current registered clinical studies span regenerative, anti-fibrotic, topical anti-inflammatory, and physical therapeutic approaches, but most remain early-stage and are primarily focused on radiation dermatitis, radiation-induced fibrosis, or selected radiation-associated soft-tissue complications. For example, an ongoing pilot study is evaluating autologous epidermal micrografts for established radiation-induced wounds, whereas a Phase II study is investigating losartan as an anti-fibrotic intervention for radiation-induced fibrosis. Physical modalities are also entering clinical evaluation; notably, a randomized feasibility study is investigating PBM for radiation-associated fibrosis and lymphedema in head and neck cancer patients. Other ongoing studies are evaluating polarized light therapy and topical diclofenac for radiation dermatitis. These studies are summarized in Table 4.

Notably, the current clinical pipeline is skewed toward prevention or treatment of acute radiation dermatitis and early fibrotic complications, whereas clinical trials specifically targeting established, refractory radiation-induced chronic ulcers remain scarce. Moreover, most registered studies rely primarily on clinical severity scores, wound characteristics, imaging parameters, or patient-reported outcomes, with relatively limited incorporation of molecular biomarkers. This gap highlights the need for biomarker-guided clinical trials that can stratify patients according to disease stage, predict therapeutic responsiveness, and provide objective measures of treatment efficacy.

### 3.4. Biomarkers for Diagnosis, Prognosis, and Therapeutic Monitoring

Despite increasing understanding of the molecular mechanisms underlying radiation-induced chronic wounds, clinically validated biomarkers for diagnosis, prognostication, and therapeutic monitoring remain lacking. Current assessment of radiation-induced skin injury primarily relies on radiation exposure history, estimated dose, clinical manifestations, and clinical grading systems such as RTOG and CTCAE. Although these tools provide a practical framework for clinical evaluation, they may not fully capture the biological heterogeneity underlying radiation-induced chronic wounds [125]. Therefore, the development of objective molecular biomarkers may improve early identification of patients at high risk for progressive and non-healing injury.

Inflammatory mediators represent potential indicators of disease activity. Persistent elevation of pro-inflammatory cytokines, including IL-1β, IL-6, and TNF-α, may reflect sustained inflammatory activation and could potentially distinguish unresolved inflammatory states from effective tissue repair. Recent clinical studies have also explored circulating cytokine profiles for predicting radiation dermatitis severity. For example, serum IL-4, IL-17, IL-1 receptor antagonist, interferon-γ, and stromal cell-derived factor-1α, together with clinical and patient-reported variables, have shown predictive value for grade ≥2 radiation dermatitis in breast cancer patients [126]. However, these biomarkers currently lack sufficient specificity for routine clinical diagnosis.

Markers of persistent molecular damage and oxidative stress may provide complementary information. Radiation-induced DNA damage can be reflected by markers such as γ-H2AX and oxidative DNA damage products such as 8-hydroxy-2′-deoxyguanosine (8-OHdG), whereas lipid peroxidation products may reflect sustained oxidative injury and ferroptotic stress. These markers are mechanistically relevant to radiation-induced tissue damage but require further clinical validation before they can be used for routine risk stratification or treatment monitoring.

Fibrosis-associated biomarkers may be particularly valuable for prognostic assessment of chronic radiation-induced wounds. Persistent activation of the TGF-β/Smad pathway, excessive extracellular matrix deposition, and dysregulated matrix turnover are central features of progressive radiation-induced fibrosis. Accordingly, TGF-β, matrix metalloproteinases (MMPs), tissue inhibitors of metalloproteinases (TIMPs), and collagen-related biomarkers may potentially reflect the transition from persistent inflammation to irreversible fibrotic remodeling. Serial assessment of these markers may also help determine whether anti-inflammatory or anti-fibrotic interventions are biologically effective.

Cellular senescence-related biomarkers may provide another layer of biological stratification. Persistent DNA damage and oxidative stress induce senescence and the senescence-associated secretory phenotype (SASP), characterized by increased expression of factors such as IL-6, IL-8, and MMPs. Therefore, senescence-associated markers, including p16^INK4a^, p21, and selected SASP components, may help identify tissues with persistent regenerative dysfunction and potentially predict poor wound healing.

The skin microbiome may also represent an emerging biomarker category for radiation-induced skin injury. Changes in microbial diversity, community composition, and the relative abundance of specific taxa have been associated with the severity and persistence of radiation dermatitis, suggesting potential utility for risk stratification and monitoring of treatment response. However, microbiome-based biomarkers remain investigational and require validation across different anatomical sites, patient populations, radiation regimens, sampling methods, and sequencing platforms. Representative candidate biomarkers across these categories are summarized in Table 5.

Crucially, while these candidate biomarkers are mechanistically relevant to radiation pathogenesis, none is sufficiently specific to radiation-induced chronic wounds when considered in isolation, as similar alterations may occur in other inflammatory, infectious, or traumatic skin conditions. Therefore, individual biomarkers should be interpreted in conjunction with relevant clinical parameters, including radiation dose and field, lesion characteristics and duration, infection status, and imaging findings. Importantly, combining biomarkers that reflect complementary pathological processes—such as inflammation, oxidative stress, cellular senescence, fibrosis, vascular dysfunction, and microbiome alterations—may provide a more robust biological signature and reduce the risk of misclassification associated with reliance on a single marker. Such multimarker approaches may ultimately improve the accuracy of diagnosis, prognostic stratification, and treatment-response monitoring.

Importantly, none of these candidate biomarkers is sufficiently specific to radiation-induced chronic wounds when considered individually. Inflammatory cytokines, oxidative stress products, senescence-associated markers, and fibrotic mediators can also be elevated in infection, aging, metabolic disorders, trauma, and other chronic inflammatory or fibrotic conditions. Similarly, alterations in the skin microbiome may reflect anatomical site, antimicrobial exposure, host characteristics, or other environmental factors rather than radiation injury itself. Therefore, these markers are more likely to provide complementary information than to serve as standalone diagnostic indicators. A multimarker panel integrating inflammatory, oxidative stress, senescence, fibrotic, vascular, and microbiome-related signatures with clinical parameters—such as radiation dose and field, wound characteristics, duration, infection status, and imaging findings—may therefore provide greater diagnostic, prognostic, and treatment-monitoring value than any single biomarker.

## 4. Conclusions and Future Perspectives

Radiation-induced chronic wounds represent one of the most severe late manifestations of radiation-induced skin injury and are characterized by persistent inflammation, microvascular dysfunction, stem cell exhaustion, and progressive fibrosis. The reciprocal interactions among these pathological processes disrupt tissue homeostasis and prevent the restoration of functional regeneration.

Although current therapies can alleviate symptoms, control infection, and promote superficial wound repair, their ability to reverse the underlying pathological alterations remains limited. Emerging approaches, including antioxidant and anti-fibrotic interventions, senolytic therapies, stem cell- and exosome-based therapies, and advanced biomaterials, have shown encouraging results in preclinical and early clinical studies. However, the translation of these emerging strategies into clinical practice remains fraught with substantial hurdles.

On the one hand, most interventions lack precision targeting of the critical pathogenic processes, failing to specifically act upon core pathological events such as persistent inflammation, senescent cell accumulation, or aberrant matrix deposition, thereby resulting in limited therapeutic efficacy or off-target effects. On the other hand, for radiation-induced wounds—which exhibit well-defined temporal phases—a single therapeutic strategy cannot possibly be applicable across all stages, and no sequential or combination regimens tailored to individual phases are currently available. Therefore, the use of advanced multi-omics technologies may be required to facilitate a shift from a cell-centric to a niche-oriented regenerative paradigm, rendering the normalization of the pathological microenvironment a prerequisite for successful tissue regeneration.

Moreover, additional challenges, including reproducibility of treatment outcomes, delivery efficiency, standardization of manufacturing protocols, long-term safety profiles, and large-scale clinical validation, continue to impede their eventual clinical implementation. Future research should focus on elucidating the spatiotemporal dynamics of radiation-induced tissue injury through advanced technologies such as single-cell sequencing, spatial transcriptomics, and multi-omics analyses, thereby identifying key therapeutic targets and molecular networks. These approaches may reveal previously unrecognized cellular states, radiation-induced memory phenotypes, and spatially restricted regenerative niches that cannot be captured by conventional bulk analyses.

In addition, shifting away from single-target interventions is imperative; future therapeutic trends must pivot toward combinatorial precision medicine that integrates epigenetic modulators (to erase fibrotic chromatin memory) with active, stimuli-responsive smart biomaterials and targeted cellular/vesicular delivery systems [127]. The establishment of validated, long-term chronic animal models and stage-specific, individualized treatment algorithms will be essential to bridge the translational gap, transforming the management of radiation-induced chronic wounds from palliative symptom control toward true functional tissue regeneration and improved patient quality of life [128].

The successful implementation of the proposed “stage-specific combination therapy” paradigm relies heavily on the identification and validation of robust predictive and prognostic biomarkers that can accurately guide personalized treatment decisions [129,130]. These biomarkers should ideally be capable of defining dynamic pathological stages—such as the acute inflammatory phase, chronic SASP-driven inflammatory phase, and fibrotic remodeling phase—and predicting therapeutic responses to specific interventions [130].

Potential candidates can be stratified into several categories: (1) circulating inflammatory cytokines (e.g., IL-6, IL-1β, TNF-α) and chemokines as potential indicators of inflammatory activity; (2) circulating biochemical indicators, including lipid peroxidation products and matrix metalloproteinases, or extracellular matrix turnover markers such as procollagen type III N-terminal peptide (PIIINP), for evaluating oxidative damage and fibrotic progression [131,132]; and (3) tissue-derived or circulating damage-associated molecular patterns (DAMPs) as indicators of active cellular injury [133].

Furthermore, advanced multi-omics technologies—including single-cell RNA sequencing and spatial transcriptomics—hold great promise for defining “stage-specific molecular signatures” by revealing dynamic cellular composition, cell–cell interactions, and spatial architecture within irradiated tissues [134].

Ultimately, the integration of these candidate molecular biomarkers with conventional clinical parameters (e.g., RTOG/CTCAE grading, imaging, and histological assessments) could provide the foundation for a comprehensive dynamic stratification algorithm. Such an algorithm could potentially guide clinicians in selecting the optimal timing and combination of anti-inflammatory, senolytic, pro-angiogenic, and anti-fibrotic interventions, facilitating the transition from empirical therapy toward precision regenerative medicine [1].

In summary, continued advances in mechanistic understanding, biomaterial engineering, and regenerative medicine are expected to transform the management of radiation-induced chronic wounds from symptom control toward functional tissue regeneration, ultimately improving patient outcomes and quality of life.

## Figures and Tables

**Table 1:** Clinical stages and characteristic features of radiation-induced skin injury.

Stage	Time After Radiation Exposure	Key Psychological Features	Typical Clinical Manifestations	Prognosis
Acute stage	Hours to 4 weeks	DNA damage, oxidative stress, acute inflammation	Erythema, edema, dry desquamation, pruritus, burning sensation; severe cases may develop blisters, erosion, exudation, and intense pain	Mild cases are reversible; moderate-to-severe cases may progress to chronicity
Subacute stage	4–12 weeks	Chronic inflammation, M1 macrophage predominance, impaired tissue repair	Pale wound bed, sparse granulation tissue, persistent crusting, telangiectasia, pigmentary changes, persistent pain	Transition from reversible to irreversible injury
Chronic stage	3–6 months	Microvascular occlusion, stem cell depletion, initiation of fibrosis	Persistent ulceration, recurrent crust formation, skin atrophy, reduced elasticity, fibrosis, indurated wound margins, susceptibility to infection	Irreversible and difficult to heal
Late stage	>6 months	Severe fibrosis, vascular occlusion, tissue necrosis	Leather-like sclerosis, contracture deformities, bone exposure, chronic infection, increased risk of malignant transformation	Extremely poor; permanent non-healing state

Note: Chronic radiation dermatitis represents a late manifestation of RISI, whereas radiation-induced chronic wounds (RICWs) refer to the persistent, non-healing ulcerative lesions that may develop in severe late-stage disease.

**Table 2:** Clinical grading of radiation-induced skin injury according to RTOG and CTCAE criteria.

Grade	Clinical Manifestations	Severity
Grade 1	Mild erythema, dry desquamation, mild pruritus	Mild; reversible
Grade 2	Moderate erythema, moist desquamation, blistering, epidermal loss, stinging pain	Moderate; reversible
Grade 3	Skin necrosis, superficial ulceration, infection	Severe; difficult to heal
Grade 4	Deep ulceration, bone exposure, extensive fibrosis, recurrent infection, malignant transformation	Very severe; irreversible

**Table 3:** Therapeutic strategy; representative approaches; primary targets/mechanisms; potential therapeutic benefits; current evidence; major limitations and challenges.

Strategy	Examples	Target	Benefits	Evidence	Challenges
Antioxidant Therapy	Fullerols; cordycepin; antioxidant hydrogels	ROS/Nrf2	↓ Oxidative stress	Preclinical/early clinical	Stability; tissue retention
Ferroptosis-targeted Therapy	Fer-1; Lip-1; DFO; DFO hydrogels	Lipid peroxidation; iron	↓ Ferroptosis, inflammation, fibrosis	Preclinical	Dose; timing; tissue specificity; safety
Anti-fibrotic Therapy	PTX + vitamin E; statins; metformin	TGF-β/Smad	↓ Fibrosis	Preclinical/clinical experience	Repair interference; limited efficacy in advanced fibrosis
Senolytic Therapy	D+Q; ABT-263; fisetin; HSP90 inhibitors	Senescent cells/SASP	↓ Chronic inflammation; ↑ regeneration	Preclinical	Timing; toxicity; target specificity
Stem cell Therapy	MSCs; modified MSCs	Paracrine signaling; angiogenesis	Tissue regeneration; immunomodulation	Preclinical/early clinical	Cell survival; standardization
Exosome Therapy	MSC-derived exosomes; exosome delivery systems	miRNAs/proteins/lipids	Cell-free regeneration	Preclinical	Clearance; targeting; standardization
Functional biomaterials	Cerium-based hydrogels; silk scaffolds; DFO-loaded hydrogels; antioxidant/oxygen-supplying hydrogels	Redox balance; ferroptosis; hypoxia; inflammation; regeneration	Localized/sustained delivery; microenvironment modulation	Preclinical	Batch variability; mechanical/degradation/release characterization; sterilization/stability; GMP and quality-control standards
Physical adjunctive Therapy	HBOT; PBM; PDT; infrared; ultrasound	Oxygenation; mitochondrial/redox signaling; perfusion	Supportive wound repair	Clinical/adjunctive	Protocol heterogeneity; dose optimization; limited disease-modifying effects

Note: ↑ indicates an increase or enhancement, whereas ↓ indicates a decrease, reduction, or inhibition.

**Table 4:** Ongoing clinical studies of therapeutic strategies for radiation-induced skin and soft-tissue injury.

ClinicalTrials.gov ID	Therapeutic Category	Intervention	Radiation-Related Condition	Study Design/ Phase	Estimated Enrollment	Primary Outcome	Status
NCT04560803	Regenerative therapy	Autologous epidermal micrografts	Radiation-induced wounds	Pilot interventional study	34	CTCAE wound grade	Recruiting
NCT05637216	Anti-fibrotic therapy	Losartan vs. placebo	Radiation-induced fibrosis	Phase II	40	Fibrosis-related outcomes	Verify at submission
NCT06708754	Physical adjunctive therapy	PBM	Radiation fibrosis/lymphedema	Randomized feasibility study	60	Soft-tissue thickness/biomarkers	Recruiting
NCT06905561	Anti-inflammatory topical therapy	Diclofenac sodium gel	Radiation dermatitis	Randomized controlled study	156	Grade ≥2 radiation dermatitis	Recruiting
NCT07600957	Physical adjunctive therapy	Polarized light therapy	Radiation dermatitis	Randomized controlled study	60	Dermal thickness/RTOG grade	Recruiting
NCT07353671	Topical/regenerative therapy	Meilian Fuxin Solution	Radiation dermatitis	Phase II/III	60	Radiation dermatitis severity	Verify latest status

**Table 5:** Candidate biomarkers for diagnosis, prognostic stratification, and therapeutic monitoring of radiation-induced chronic wounds.

Biomarker Category	Candidate Biomarkers	Potential Clinical Relevance	Key Limitations/Specificity
Inflammatory	IL-1β, IL-6, TNF-α	Inflammatory activity; treatment response	Also elevated in infection and other inflammatory conditions
DNA damage/oxidative stress	γ-H2AX, 8-OHdG, lipid peroxidation products	Persistent radiation-associated cellular stress	Not specific to radiation; also induced by other sources of oxidative/DNA damage
Cellular senescence/SASP	p16^INK4a^, p21, IL-6, IL-8, MMPs	Senescence burden; impaired regeneration	May increase with aging and other chronic diseases
Fibrosis/ECM remodeling	TGF-β, MMPs, TIMPs, collagen-related markers	Fibrotic progression; prognosis	Altered in non-radiation-related fibrotic disorders
Vascular dysfunction/hypoxia	HIF-1α, vascular-related markers	Tissue hypoxia; impaired angiogenesis	Reflect downstream tissue dysfunction rather than radiation-specific injury
Skin microbiome	Microbial diversity, community composition, specific taxa	Risk stratification; prognosis; treatment monitoring	Strongly affected by anatomical site, antimicrobial exposure, host and environmental factors

## Data Availability

No new data were created or analyzed in this study. Data sharing is not applicable to this article.

## Abbreviations

Ang-1, angiopoietin-1; ATP, adenosine triphosphate; CTCAE, Common Terminology Criteria for Adverse Events; CXCL2, CXC chemokine ligand 2; COX, cytochrome c oxidase; p16^INK4a^, Cyclin-dependent kinase inhibitor2A(p16^INK4a^); DAMPs, damage-associated molecular patterns; DDR, DNA damage response; DSBs, DNA double-strand breaks; D+Q, dasatinib and quercetin; ECM, extracellular matrix; EGF, epidermal growth factor; EpSCs, epidermal stem cells; FGF, fibroblast growth factor; GPX4, glutathione peroxidase 4; GSH, glutathione; γ-H2AX, Gamma-H2AX (phosphorylated histone H2AX); HBOT, hyperbaric oxygen therapy; HIF-1α, hypoxia-inducible factor-1α; HMGB1, high-mobility group box 1; HO-1, heme oxygenase-1; HSP90, heat shock protein 90; 8-OHdG, 8-hydroxy-2′-deoxyguanosine; IL-1β, interleukin-1β; IL-1Ra, Interleukin-1 receptor antagonist; IL-4, Interleukin-4; IL-6, interleukin-6; IL-10, interleukin-10; IL-17, Interleukin-17; IL-33, interleukin-33; IFN-γ, Interferon-gamma; MAPK, mitogen-activated protein kinase; MCP-1, monocyte chemoattractant protein-1; MMPs, matrix metalloproteinases; MSCs, mesenchymal stem cells; NETs, neutrophil extracellular traps; NF-κB, nuclear factor-κB; NLRP3, NOD-like receptor family pyrin domain-containing 3; Nrf2, nuclear factor erythroid 2-related factor 2; PAMPs, pathogen-associated molecular patterns; PBM, photobiomodulation; PDGF-AA, platelet-derived growth factor AA; PDT, photodynamic therapy; PIIINP, procollagen type III N-terminal peptide; PRRs, pattern recognition receptors; RAGE, receptor for advanced glycation end-products; RISI, radiation-induced skin injury; ROS, reactive oxygen species; RTOG, Radiation Therapy Oncology Group; SASP, senescence-associated secretory phenotype;SDF-1α, Stromal cell-derived factor-1 alpha; SLC7A11, System L amino acid transporter light chain subunit; TGF-β, transforming growth factor-β; THBS1, thrombospondin-1; TIMPs, tissue inhibitors of metalloproteinases; TLR4, Toll-like receptor 4; TNF-α, tumor necrosis factor-α; TREM2, triggering receptor expressed on myeloid cells 2; VEGF, vascular endothelial growth factor.
